# Impact of arm position compared to tourniquet and general anesthesia on peripheral vein width in supine adult patients: a prospective, monocentric, cross-sectional study

**DOI:** 10.1186/s12871-024-02765-6

**Published:** 2024-10-22

**Authors:** Christian Berger, Philipp Brandhorst, Elena Asen, Sven Grallert, Sascha Treskatsch, Moritz Weigeldt

**Affiliations:** 1 Department of Anaesthesiology and Intensive Care Medicine, Charité - Universitätsmedizin Berlin, Corporate Member of Freie Universität and Humboldt Universität zu Berlin, Campus Benjamin Franklin, Hindenburgdamm 30, Berlin, 12203 Germany; 2 Department of Anaesthesiology, Intensive Care-, Emergency- and Pain-Medicine, Evangelical Hospital Herne, Westring 24, Herne, 44623 Germany; 3 Department of Intensive Care and Emergency Medicine, Helios Hospital Emil Von Behring, Walterhöferstraße 11, Berlin, 14165 Germany

**Keywords:** Ultrasound, Ultrasound guiding, Venous access, Patient safety, Vein size, Vein width

## Abstract

**Background:**

IV access is a commonly performed procedure that is often taught based on tradition rather than evidence. The effect of arm retroflexion on vein width, either alone or in combination with a tourniquet or general anesthesia (GA), remains unclear. In this case, the sonographically measured vein width is a surrogate parameter for the success of the puncture.

**Methods:**

Prospective, cross-sectional study involving 57 patients scheduled for surgery in general anesthesia. We analyzed the impact of arm retroflexion, tourniquet, general anesthesia, and their combinations on the antebrachial veins in supine patients by ultrasound. Measurements were taken awake and during general anesthesia, each with and without the application of a tourniquet, and in three different arm positions (0°, 30°, and max° retroflexion) each. Data are presented as median and interquartile range [IQR].

**Results:**

Tourniquet application (AT) had the greatest single effect on Cubital vein outer diameter compared to the baseline value of all measures (3.9 mm [3.4–5.1]; 4.8 mm [4.1–5.7], *P* = 0.001, *r* = 0.515). This effect was surpassed by the combination of AT and GA (5.1 mm [4.6–6.6], *P* = 0.001, *r* = 0.889). In contrast, retroflexion alone did not result in an increase at either 30° (4.2 mm [3.7–5.1], *p* = 1.0, *r* = 0.12) or max° (4.2 mm [3.6–4.9], *p* = 0.72, *r* = 0.23). With GA and AT, no further enlargement was measurable by 30° (5.4 mm [4.6–6.6], *p* = 1.0, *r* = 0.15) or max° (5.4 mm [4.6–6.6], *p* = 1.0, *r* = 0.07) retroflexion compared to GA-AT-0° (5.1 mm [4.6–6.6], *p* = 1.0, *r* = 0.15).

**Conclusions:**

This study provides evidence that retroflexion of the arm in supine patients, whether alone or in addition to a tourniquet or general anesthesia, does not have any additional effect on vein width as a surrogate parameter for successful IV success. It shows for the first time that general anesthesia effectively increases vein diameter.

**Trial registration:**

DRKS00029603 (date of registration 07.07.2022).

**Supplementary Information:**

The online version contains supplementary material available at 10.1186/s12871-024-02765-6.

## Background

Peripheral intravenous (IV) access for rapid and safe administration of necessary medications is a fundamental medical procedure in clinical practice, especially in anesthesia as well as in emergencies. Delayed insertion due to multiple puncture attempts delays drug administration and can be painful and distressing for patients [1]. Multiple punctures are associated with complications like nerve damage, hematoma, and higher medical costs [2]. Therefore, measures increasing vein size should be applied for IV access, as a larger vein diameter is associated with a higher success rate of peripheral vein cannulation [3–5].

Applying a tourniquet for increasing venous filling by venous pooling, as well as tapping of veins, hand pumping, and application of heat, are known measures for increasing vein size [6–10]. The patient´s posture as well as lowering the arm below the heart´s level further seems to affect peripheral vein diameter but with inconsistent results [11, 12]. IV access is mostly performed in the supine position in emergencies and during general anesthesia (GA). However, vein enlarging measures have been poorly studied under these conditions, and IV puncture is taught out of tradition and habit rather than evidence. Despite this lack of evidence, there are recommendations to position the arm below the heart regardless of patient´s posture [13, 14]. It is currently unclear if this maneuver has a positive effect on vein size in a supine position. Nevertheless, it is performed in both, awake and anesthetized patients in clinical practice. Furthermore, retroflexion has not yet been evaluated in conjunction with other measures like GA or Tourniquet. Thus, the question of whether retroflexion in the shoulder joint of supine patients, either alone or in combination with GA or tourniquet, effectively increases IV puncture success remains unresolved. This is particularly crucial to address when additionally considering the potentially harmful effects of arm retroflexion under GA, such as stretching of the plexus may lead to relevant nerve damage [15, 16]. Therefore, we conducted this prospective cross-sectional trial, analyzing the impact of upper limb retroflexion, both independently and in conjunction with tourniquet and GA on peripheral vein size, as a surrogate of IV puncture success.

## Methods

### Study design

This study was a monocentric, prospective, cross-sectional trial at a tertiary hospital. Ethical approval was provided by the Ethikkommission der Charité—Universitätsmedizin Berlin, Germany (Ethical Committee N° EA4/102/22). All patients were checked for eligibility and gave their written informed consent. The trial was registered prior to patient enrollment at WHO International Clinical Trials Registry Platform and German Clinical Trial Register (DRKS00029603, date of registration 07.07.2022). The study protocol was carried out in accordance with the Declaration of Helsinki and this manuscript adheres to the applicable CONSORT guidelines.

### Patients and setting

The trial was performed from the 7th of July 2022 to the 13th of September 2022. Adult patients meeting ASA Classification I or II criteria scheduled for elective surgery in general anesthesia were enrolled in this study. Patients with disease or surgery of the upper extremities, shoulders, or thorax, and patients who met any of the following criteria were excluded: systemic vascular disease, recent thrombotic events, local vascular disease of the upper extremities, pre-existing cardiac disease associated with systolic or diastolic dysfunction, venous puncture on the limb to be measured within two weeks before examination, frequent venous punctures in the history or vasoactive concomitant medication.

**Fig. 1:**
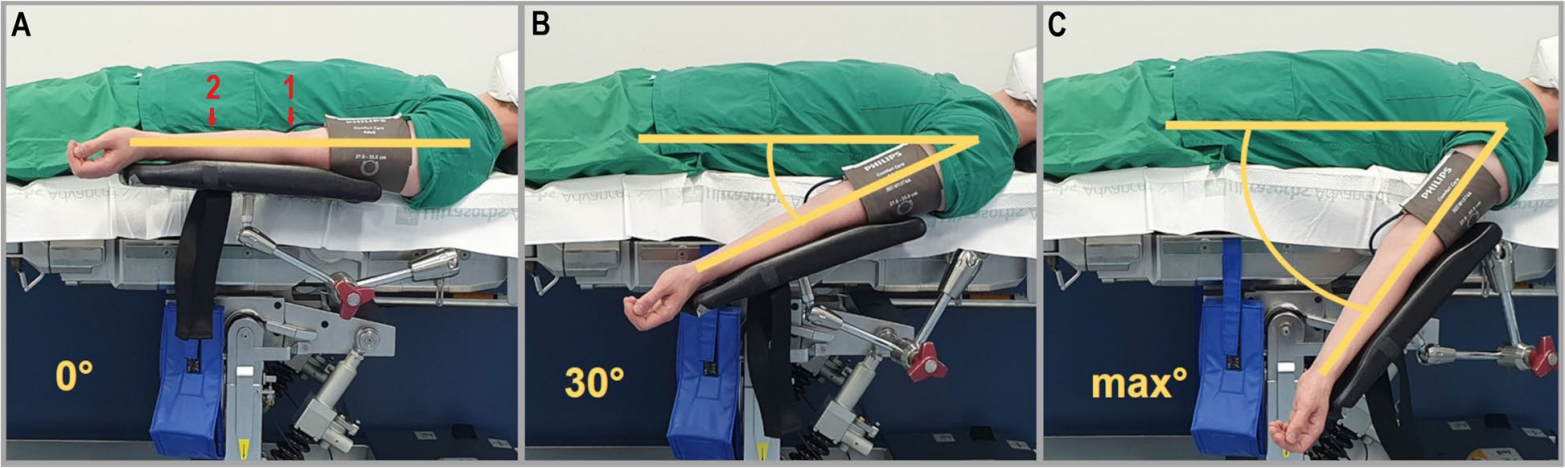
Figure showing the positioning of the arm in relation to the shoulder joint. **a** 0° position, 1 shows the measuring point of the cubital vein, and 2 is the measuring point of the cephalic vein. **b** 30° retroflexion position. **c** maximum retroflexion position (median 75° [Interquartile range 65–90])

All procedures and measurements were performed in a tempered (19–21 °C) anesthesia induction room of a central operating theatre area. Patients were placed in a supine position on an operating table with armrests padded underneath and fixable at respective degree settings in the shoulder joint during the whole procedure (Fig. 1).

### Measurements

All measurements were performed on the cubital vein (CuV) and cephalic vein (CeV) on the nondominant arm using a high-frequency linear ultrasound probe (8–18 MHz, ultrasound device: Vivid iq, GE Healthcare, Chicago, IL, USA). Optimal measurement positions were identified in the fossa cubiti and at the middle of the forearm by ultrasound (Fig. 1a). To ensure comparability, the position was marked and all subsequent measurements were taken at these markings. To avoid potential changes in vein geometry and size, the probe was placed gently on the skin to obtain an image without applying additional pressure. Each set of measurements included vein circumference (CI) and vein diameter in the out-of-plane probe position as well as vein diameter in the in-plane probe position to ensure accurate 2D measurements. CI was measured by tracing the edges and all diameters were measured from anterior to posterior (Supplemental Fig. 1).

All measurements were performed under the following patients’ conditions in consecutive order: 1) patient awake without tourniquet (awake-NT), 2) patient awake with tourniquet (awake-AT), 3) patient in GA without tourniquet (GA-NT), and 4) patient in GA with tourniquet (GA-AT). Within each condition, different levels of retroflexion (0°, 30°, and max°; Fig. 1a-c) were applied in a randomized order.

The patients waited in their respective positions for 60 s before the corresponding measurement was taken. After each measurement, patients rested in a supine position without a tourniquet and in 0° arm retroflexion for 2 min to ensure consistency in the starting position for each subsequent condition and arm position during measurement.

The maximum possible retroflexion (max°) was determined in advance by slowly rotating the patient´s outstretched arm passively backward from the horizontal axis. As soon as the maximum possible retroflexion was reached or the patient indicated paresthesia or pain, the arm was slightly returned from retroflexion until there was no more discomfort. The thus determined degree of retroflexion [°] corresponded to the max° position for each patient.

A tourniquet was applied using a 13.5 cm wide blood pressure cuff, placing it 2–3 cm above the cubital fossa and inflating it to 60 mmHg. This setting was selected because it demonstrated the greatest impact on vein enlargement and decreased compressibility when compared to other tourniquet techniques [17]. The optimal result was achieved when the cuff was inflated to a pressure of 60 mmHg and applied for 30–60 s. Following this period, no further increase in vein size was observed [10].

According to these previous findings, our patients remained in each condition for 60 s before measurements were performed and the strong effect of tourniquet application was confirmed in our work.

Apart from the study-related procedures described above, no further measures were taken on the nondominant arm throughout the whole study period. Monitoring for anesthesia and IV access were established in the dominant arm before the study measurement. Peripheral oxygen saturation as well as heart rate were measured continuously. Blood pressure was measured initially before awake measurements and with the beginning of GA induction in 2.5-min intervals on the dominant arm. Induction and maintenance of general anesthesia were performed according to institutional standard operating procedures. Induction was performed with an opioid followed by a bolus of propofol, maintenance with propofol or sevoflurane.

### Statistics

For analyzing measures to maximize vein size within a study cohort, a sample size calculation was performed in advance with a two-sided significance level of 5% and a power of 80%. Based on the previously reported effects of tourniquet application which range from 0.14–0.87 mm, with a maximum standard deviation of 1.3 mm, a mean change of 0.505 mm in vein diameter was assumed [12]. The calculation of the sample size yielded 54 patients for our study. To account for a potential dropout rate of 10%, the study was planned with a final total of 60 patients.

Data processing and analysis were performed using IBM SPSS Statistics (version 25; IBM, Armonk, NY, USA). Data are presented as median (Interquartile range [IQR]). Differences between measurements were evaluated using a paired Friedman test with Bonferroni-correction for multiple comparisons. Significance was set at *P* < 0.05, also for Bonferroni-correction after mathematical adjustment was applied. The effect size for non-parametric testing was estimated using a z-statistic by calculating correlation coefficient *r*, with > 0.1 representing a small, > 0.3 a medium, and > 0.5 a large strength of association, according to the recommendations of Cohen [18]. To analyze the accuracy of the measurements, the intra-class correlation coefficient was calculated between in-plane and out-of-plane measurements.

Patient demographics and hemodynamic/anesthesia data were reported as qualitative data and percentage or median [IQR], as applicable. Demographic as well as hemodynamic data and ventilation pressure settings were reported for transparency of the study cohort. No further statistical analyses were performed on this data.

Only patients with complete data sets were included in the final data analyses.

## Results

**Fig. 2:**
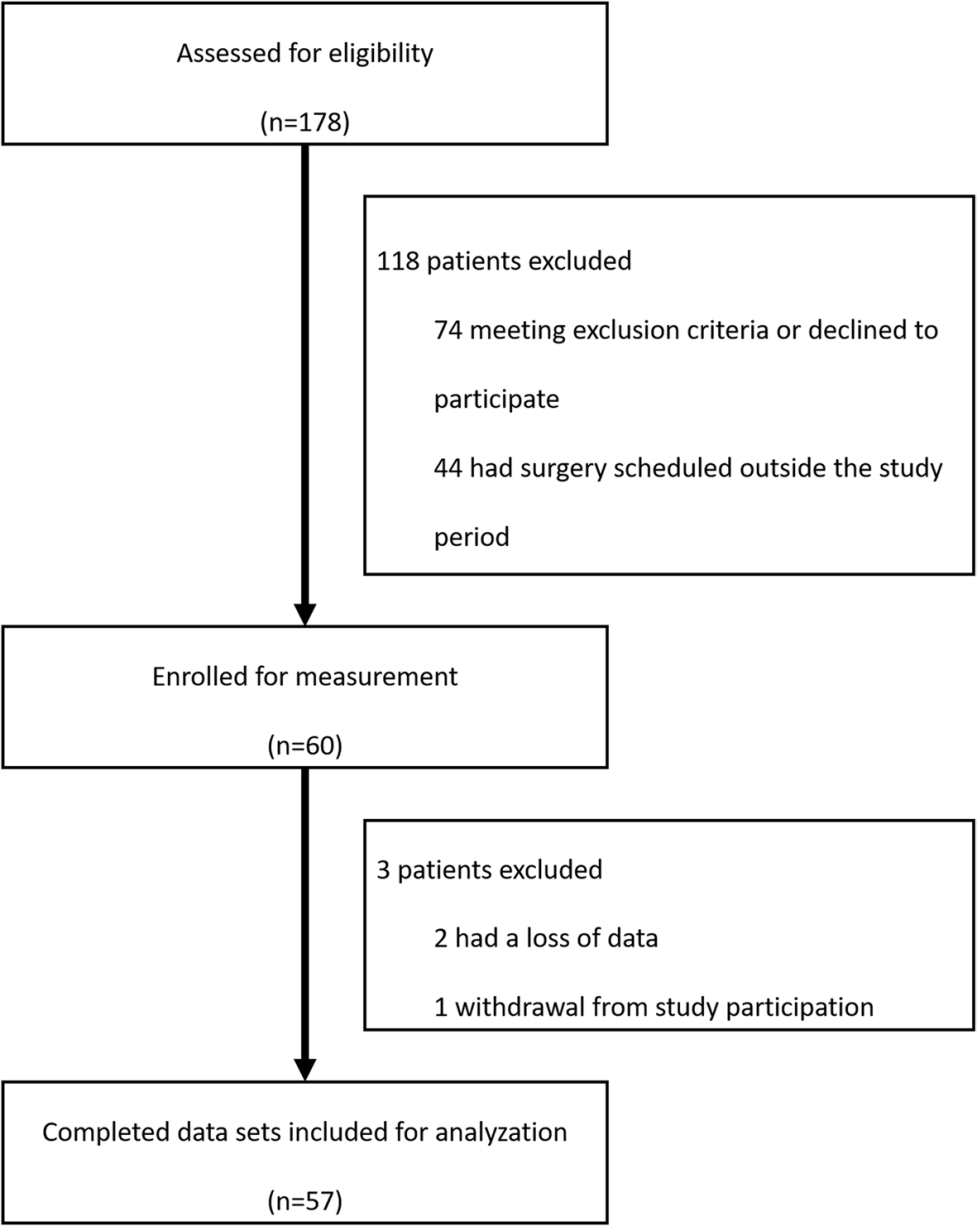
Consort flowchart

For the final analysis, 57 patients with complete data sets were included (Fig. 2). Patients´ characteristics are presented in Table 1. Detailed hemodynamic data and ventilator settings are presented in Supplemental Table 1. The mean peak respiratory pressure was 15 [14–17] mbar and the mean positive end-expiratory pressure was 5 [5–6] mbar.After awake measurements, GA was induced with 200 [145–200] mg propofol in all cases and supplemented with sufentanil/fentanyl in 32 (56%) or remifentanil in 25 (44%) cases. Maintenance of general anesthesia was performed with propofol in 37 (65%) and sevoflurane in 20 (35%) cases. 100 [50–230] ml of balanced isotone electrolyte solution was administered from induction of general anesthesia up to final measurements.

**Table 1:** Patient characteristics

Variables	Total study sample (*n* = 57)
Age, years (median [IQR])	40 [27–51]
Body mass index, kg/m^2^ (median [IQR])	24.2 [22.2–26.9]
Sex (female), n (%)	30 (53%)
Right handed, n (%)	55 (97%)
max° retroflexion, degree (median [IQR])	75 [65–90]

*Abbreviation*: *IQR* Interquartile range, max° retroflexion, Maximum retroflexion in the shoulder joint

### Effect of retroflexion, general anesthesia and tourniquet

**Fig. 3:**
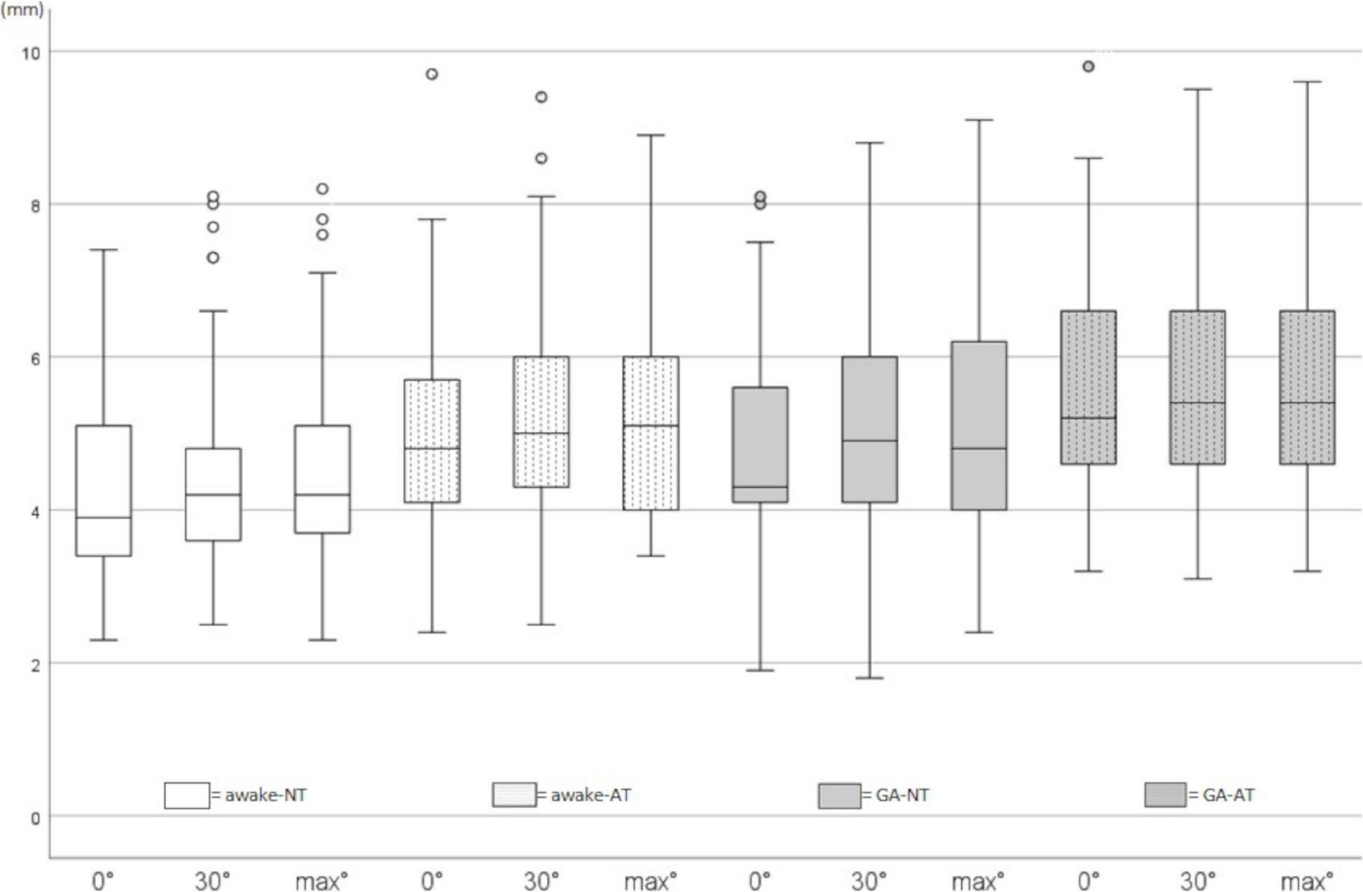
Boxplots showing Cubital Vein out-of-plane measured diameter (CuV-OD) in millimeter [mm] for 0°-30°-max° retroflexion, eeach measured in awake without tourniquet applied (awake-NT), awake with applied tourniquet (awake-AT), general anesthesia without tourniquet applied (GA-NT) and general anesthesia with applied tourniquet (GA-AT)

**Fig. 4:**
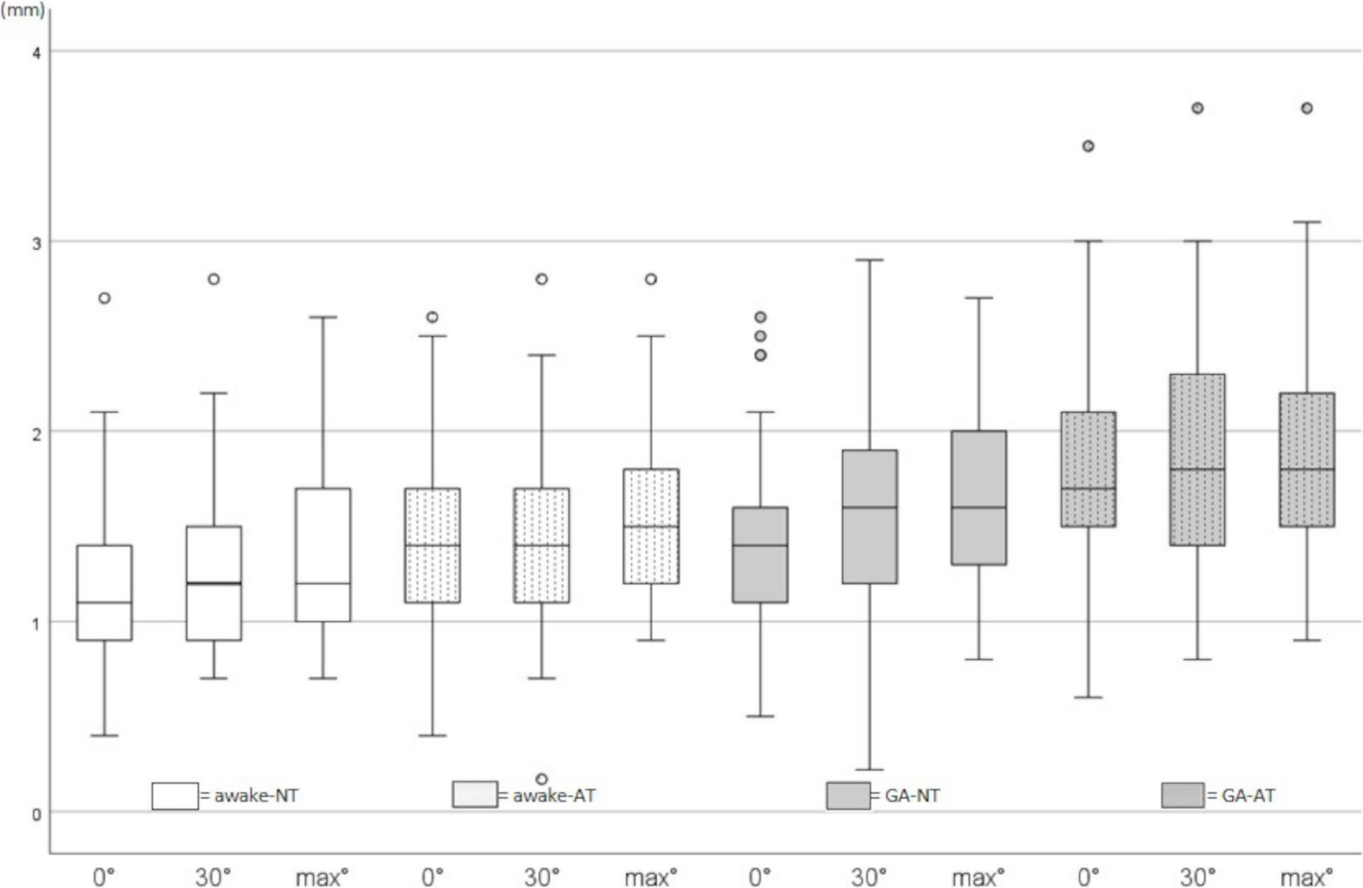
Boxplots showing the cephalic vein out-of-plane measured diameter (CeV-OD) in millimeter [mm] for 0°-30°-max° retroflexionm, each measured in awake with no tourniquet applied (awake-NT), awake with applied tourniquet (awake-AT), general anesthesia with no tourniquet applied (GA-NT) and general anesthesia with applied tourniquet (GA-AT)

A retroflexion of 30° or max° retroflexion in the shoulder joint was not associated with an increase in the CuV out-of-plane diameter compared to 0° within all conditions (Fig. 3, Table 2). CeV out-of-plane diameters did not show any effect of retroflexion, except for max° retroflexion compared to 0° in GA-NT (Fig. 4, Table 2).

**Table 2:** Results for cubital and cephalic vein out of plane diameter

Measuring condition		0° Median [IQR]	*P* value/r vs awake-NT-0°	30° Median [IQR]	*P* value/r 0° vs 30°	Max° Median [IQR]	*P* value/r 0° vs max°
CuV, awake	NT, mm	3.9 [3.4–5.1]	-	4.2 [3.7- 5.1]	1.0/0.12	4.2 [3.6–4.9]	0.720/0.23
	AT, mm	4.8 [4.1–5.7]	**0.001/0.515**	5.1 [4.0- 6.1]	1.0/0.17	5.0 [4.3–6.1]	1.0/0.171
CuV, GA	NT, mm	4.3 [4.1–5.8]	**0.030/0.315**	4.8 [4.0- 6.3]	1.0/0.216	4.9 [4.1–6.1]	0.930/0.22
	AT, mm	5.1 [4.6–6.6]	**0.001/0.889**	5.4 [4.6- 6.7]	1.0/0.15	5.4 [4.6–6.6]	1.0/0.070
CeV, awake	NT, mm	1.1 [0.9–1.4]	-	1.2 [0.9- 1.5]	1.0/0.145	1.2 [1.0–1.7]	0.456/0.242
	AT, mm	1.4 [1.1–1.7]	**0.001/0.417**	1.4 [1.1–1.7]	1.0/0.058	1.5 [1.2–1.9]	1.0/0.180
CeV, GA	NT, mm	1.4 [1.1–1.7]	0.053/0.300	1.6 [1.2–1.9]	0.229/0.261	1.6 [1.3–2.0]	**0.002/0.375**
	AT, mm	1.7 [1.5–2.1]	**0.001/0.866**	1.8 [1.4–2.3]	1.0/0.120	1.8 [1.5–2.2]	1.0/0.145

*P* value/r considering *P* < 0.05 as significant and *r* > 0.1 a small, > 0.3 a medium, and > 0.5 a large strength of association

*Abbreviations*: *AT* Applied tourniquet, *CeV* Cephalic vein, *CuV* Cubital vein, *GA* General anesthesia, *IQR* Interquartile range, *NT* Without applied tourniquet

The application of a tourniquet resulted in a significant increase in the out-of-plane diameter of both CuV and CeV, with a greater effect size observed when used in combination with GA (Figs. 3 and 4).

Application of GA alone, without a tourniquet, had a significant but reduced effect on CuV out-of-plane diameter compared to tourniquet application in GA (CuV out-of-plane diameter 0° awake-AT compared to 0° GA-NT: *p* = 0.001, *r* = 0.574; Table 2).

The intra-class correlation coefficient for CuV-awake out-of-plane and in-plane measurements was found to be 0.952 [0.919–0.972] and the CeV-awake out-of-plane and in-plane measurement comparison revealed a coefficient of 0.840 [0.728- 0.905].

Analyzation of CuV-CI and CeV-CI measurements revealed comparable results for retroflexion, Tourniquet, and GA (Supplemental Tab. 2–3, Supplemental Fig. 2–3).

## Discussion

Our findings indicate that using a tourniquet and/or general anesthesia can effectively increase peripheral vein width. However, retroflexion of 30° or max° does not have any effect on vein width in supine patients. Previous studies have not thoroughly analyzed the impact of upper limb retroflexion with tourniquet application and its effect on vein width while in the supine position. Additionally, this is the first study to include patients in both awake and anesthetized states in its analysis.

Only one earlier work by Cappelletti et al. showed an increase in the diameter and area of peripheral cephalic and basilic veins in healthy, awake subjects in the supine position with the arm dangling at a 90° angle compared with 0° [19]. This contrasts sharply with our findings, possibly due to varying study conditions.

First, our maximum position was less retroflexed, as our awake evaluation of possible maximal retroflexion indicated that the majority of patients were unable to achieve 90° retroflexion without paresthesia. Considering the potentially increased risk of plexus affection, especially in anesthetized patients, we avoided a dangling arm [15].

However, our data does not allow us to eliminate the possibility that the discrepancy between an arm bent backward by 75° and an arm dangling at 90° may have resulted in different vein width outcomes.

Secondly, the measurements were conducted in a more proximal position than in our CeV measurements [15]. Additionally, compared to our results, an evaluation by Yamagami et al. demonstrated larger baseline diameter, but these measurements were taken at different sites along the CeV [11]. One possible explanation for these discrepancies in baseline widths is the lower consistency of the measurement points in previous evaluations [6, 8].

Furthermore, heat affects vein width, which is reflected by larger baseline results in studies with slightly higher OR temps of 22–24 °C, compared to our setting [20, 21]. Besides room temperature, local warming or tapping are other, previously reported measures that can influence vein size [6, 8, 20, 22]. We avoided them to ensure the comparability of the measures evaluated.

Our in-plane measurements agree with the out-of-plane measurements and can thus be considered as a validation of our measurement procedures (Supplemental Fig. 2–3). This good correlation between the different measurement approaches indicates that differences in skin pressure with the probe, which could lead to possible vein deformation, were effectively avoided.

In contrast to many previous works that analyzed the effects on vein width only in healthy volunteers, our study is the first to examine patients before and after induction of anesthesia [6, 8, 10–12, 19, 20, 22]. GA, and especially propofol, is known to decrease peripheral vascular resistance after induction [23]. The findings of our research confirm the effects of general anesthesia on the dimensions of peripheral veins. Additionally, further retroflexion did not result in any significant increase in size.

In contrast to healthy volunteers, all our patients were fasted before surgery, which may affect venous filling, especially since fasting has been discussed as a cause of difficult venipuncture [24, 25]. Previous research revealed controversial results regarding venous filling and reduced fluid intake during fasting. One study showed an increase in the cross-sectional diameter of the vena cava inferior after oral hydration in fasted patients [26]. Sharp et al. demonstrated a reduction in forearm vein diameter after rehydration in fasting patients and discussed sympathetic nervous system activation as a possible cause [20]. Given these published controversial results, we administered fluids only at induction of general anesthesia and mostly in a “mild” form of less than 2 ml/kg body weight. Positive pressure ventilation can also act like a Valsalva maneuver and thus affect vein width [27]. In our study, the respiratory settings were within ranges considered normal, without particularly increased respiratory pressures, and were not altered during measurements for individual patients.

We chose to evaluate patients in the supine position because it is the usual position for IV access before general anesthesia as well as in pre- and intrahospital emergencies [5, 28]. It has also been demonstrated that the size of the veins appears to be larger in the supine position than in the seated position [11].

Furthermore, all measurements in our work were performed within a short period with comparable baseline conditions, which underlines the pronounced effect of tourniquet application.

### Limitations of the study

It is known that vein width is influenced by gender [29], which was not explicitly analyzed in this work. Nevertheless, male and female participants were equally distributed in this work.

For patient comfort and to avoid impairment of vein width by multiple punctures, cannula insertion at the measured extremity was omitted. IV puncture success was not measured and can only be estimated by vein width [3, 4].

## Conclusion

The application of a tourniquet had the greatest effect on the dilation of peripheral veins in supine patients, especially in anesthetized conditions. Additionally, our findings showed a strong increase in vein widths after GA induction. In contrast, arm retroflexion, alone or in combination, showed no further effects on vein width.

## Supplementary Information

Supplementary Material 1: Supplemental Fig. 1. Ultrasound measurement setting from anterior to posterior (AP). Each set of measurements included vein circumference (CI) and vein diameter in the out-of-plane probe position (OD) as well as vein diameter in the in-plane probe position (ID) to ensure accurate 2D measurements. Supplemental Fig. 2. Boxplots showing Cubital Vein circumference (CuV-CI) [mm] for 0°—30°—max° retroflexion. Each measured in awake without tourniquet applied (awake-NT), awake with applied tourniquet (awake-AT), general anesthesia without tourniquet applied (GA-NT) and general anesthesia with applied tourniquet (GA-AT). Table showing statistics presented as P/r for every comparison. Considering *P* < 0.05 as significant and *r* > 0.1 a small, > 0.3 a medium, and > 0.5 a large strength of association. Supplemental Fig. 3. Showing Boxplots Cephalic Vein circumference (CeV-CI) [mm] for 0°—30°—max° retroflexion. Each measured in awake without tourniquet applied (awake-NT), awake with an applied tourniquet (awake-AT), general anesthesia without tourniquet applied (GA-NT) and general anesthesia with an applied tourniquet (GA-AT). Table showing statistics presented as P/r for every comparison. Considering *P* < 0.05 as significant and *r* > 0.1 a small, > 0.3 a medium, and > 0.5 a large strength of association.

Supplementary Material 2.

## Data Availability

Data is provided within the manuscript or supplementary information files, the complete data set will be provided by the corresponding author upon request.

## Abbreviations

AT: Tourniquet application
CeV: Cephalic vein
CI: Circumference
CuV: Cubital vein
GA: General anesthesia
IQR: Interquartile range
IV: Intravenous
NT: Without Tourniquet
