# Supplementary figures and images for: Impact of arm position compared to tourniquet and general anesthesia on peripheral vein width in supine adult patients: a prospective, monocentric, cross-sectional study
Source: BMC Anesthesiol. 2024 Oct 22;24:379. doi: 10.1186/s12871-024-02765-6 (PMC11494795; doi:10.1186/s12871-024-02765-6)

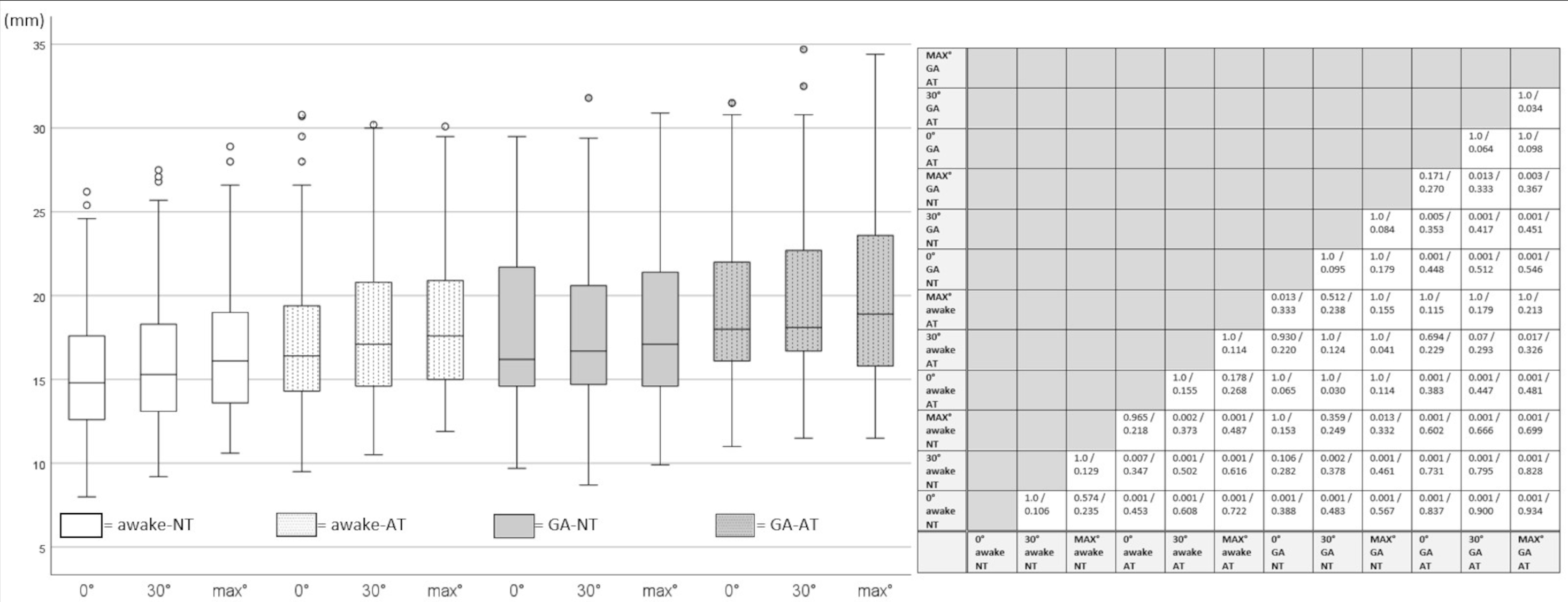

Supplement: Supplementary file 1 — Supplementary Material 1: Supplemental Fig. 1. Ultrasound measurement setting from anterior to posterior (AP). Each set of measurements included vein circumference (CI) and vein diameter in the out-of-plane probe position (OD) as well as vein diameter in the in-plane probe position (ID) to ensure accurate 2D measurements. Supplemental Fig. 2. Boxplots showing Cubital Vein circumference (CuV-CI) [mm] for 0°—30°—max° retroflexion. Each measured in awake without tourniquet applied (awake-NT), awake with applied tourniquet (awake-AT), general anesthesia without tourniquet applied (GA-NT) and general anesthesia with applied tourniquet (GA-AT). Table showing statistics presented as P/r for every comparison. Considering P < 0.05 as significant and r > 0.1 a small, > 0.3 a medium, and > 0.5 a large strength of association. Supplemental Fig. 3. Showing Boxplots Cephalic Vein circumference (CeV-CI) [mm] for 0°—30°—max° retroflexion. Each measured in awake without tourniquet applied (awake-NT), awake with an applied tourniquet (awake-AT), general anesthesia without tourniquet applied (GA-NT) and general anesthesia with an applied tourniquet (GA-AT). Table showing statistics presented as P/r for every comparison. Considering P < 0.05 as significant and r > 0.1 a small, > 0.3 a medium, and > 0.5 a large strength of association. [file 12871_2024_2765_MOESM1_ESM.zip › CUVE_supplemental_Figures_2_BMCA.png]

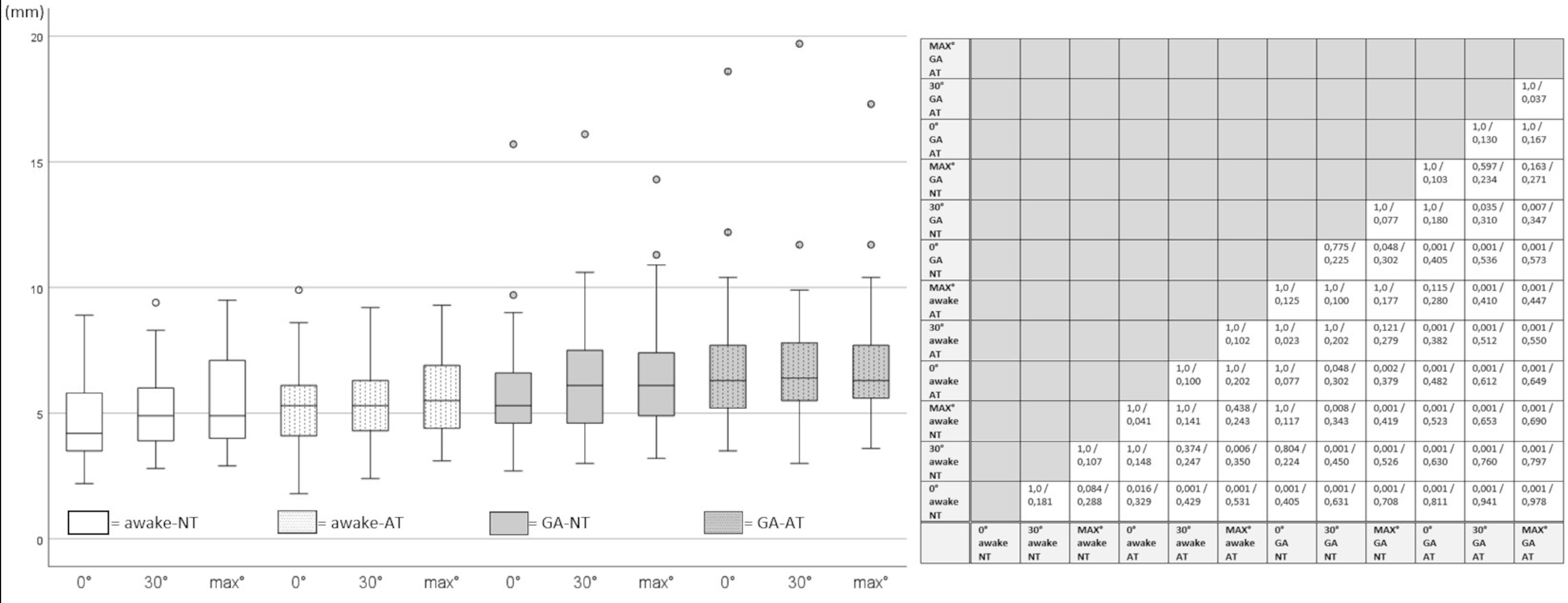

Supplement: Supplementary file 1 — Supplementary Material 1: Supplemental Fig. 1. Ultrasound measurement setting from anterior to posterior (AP). Each set of measurements included vein circumference (CI) and vein diameter in the out-of-plane probe position (OD) as well as vein diameter in the in-plane probe position (ID) to ensure accurate 2D measurements. Supplemental Fig. 2. Boxplots showing Cubital Vein circumference (CuV-CI) [mm] for 0°—30°—max° retroflexion. Each measured in awake without tourniquet applied (awake-NT), awake with applied tourniquet (awake-AT), general anesthesia without tourniquet applied (GA-NT) and general anesthesia with applied tourniquet (GA-AT). Table showing statistics presented as P/r for every comparison. Considering P < 0.05 as significant and r > 0.1 a small, > 0.3 a medium, and > 0.5 a large strength of association. Supplemental Fig. 3. Showing Boxplots Cephalic Vein circumference (CeV-CI) [mm] for 0°—30°—max° retroflexion. Each measured in awake without tourniquet applied (awake-NT), awake with an applied tourniquet (awake-AT), general anesthesia without tourniquet applied (GA-NT) and general anesthesia with an applied tourniquet (GA-AT). Table showing statistics presented as P/r for every comparison. Considering P < 0.05 as significant and r > 0.1 a small, > 0.3 a medium, and > 0.5 a large strength of association. [file 12871_2024_2765_MOESM1_ESM.zip › CUVE_supplemental_Figures_3_BMCA.png]

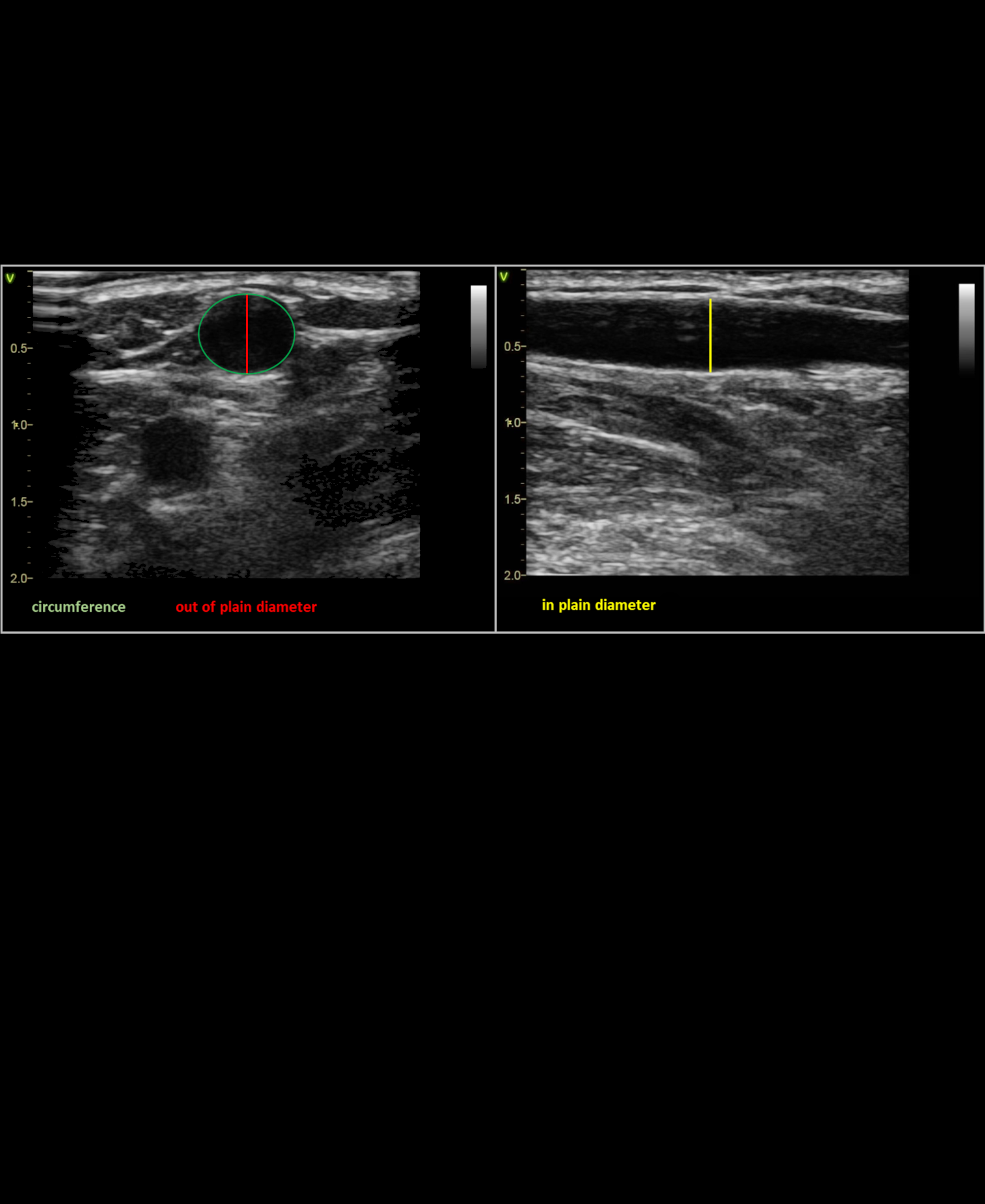

Supplement: Supplementary file 1 — Supplementary Material 1: Supplemental Fig. 1. Ultrasound measurement setting from anterior to posterior (AP). Each set of measurements included vein circumference (CI) and vein diameter in the out-of-plane probe position (OD) as well as vein diameter in the in-plane probe position (ID) to ensure accurate 2D measurements. Supplemental Fig. 2. Boxplots showing Cubital Vein circumference (CuV-CI) [mm] for 0°—30°—max° retroflexion. Each measured in awake without tourniquet applied (awake-NT), awake with applied tourniquet (awake-AT), general anesthesia without tourniquet applied (GA-NT) and general anesthesia with applied tourniquet (GA-AT). Table showing statistics presented as P/r for every comparison. Considering P < 0.05 as significant and r > 0.1 a small, > 0.3 a medium, and > 0.5 a large strength of association. Supplemental Fig. 3. Showing Boxplots Cephalic Vein circumference (CeV-CI) [mm] for 0°—30°—max° retroflexion. Each measured in awake without tourniquet applied (awake-NT), awake with an applied tourniquet (awake-AT), general anesthesia without tourniquet applied (GA-NT) and general anesthesia with an applied tourniquet (GA-AT). Table showing statistics presented as P/r for every comparison. Considering P < 0.05 as significant and r > 0.1 a small, > 0.3 a medium, and > 0.5 a large strength of association. [file 12871_2024_2765_MOESM1_ESM.zip › CUVE_supplemental_Figure_1_BMCA_revision.png]
