# Supplementary material for: Impact of arm position compared to tourniquet and general anesthesia on peripheral vein width in supine adult patients: a prospective, monocentric, cross-sectional study
Source: BMC Anesthesiol. 2024 Oct 22;24:379. doi: 10.1186/s12871-024-02765-6 (PMC11494795; doi:10.1186/s12871-024-02765-6)
Supplement: Supplementary file 2 — Supplementary Material 2. [file 12871_2024_2765_MOESM2_ESM.zip › CUVE_suppl_table_1_BMCA.docx]

**SUPPLEMENTAL TABLES**

Supplemental Tab.1 Hemodynamic data and ventilation pressure settings before measurements in the awake and anesthetized state.

| Parameters | Awake, median [IQR] | GA, median [IQR] |
| --- | --- | --- |
| SBP (mmHg) | 119 [112-128] | 99 [91-109] |
| DBP (mmHg) | 75 [69-82] | 60 [54-68] |
| MAP (mmHg) | 89 [82-96] | 72 [66-79] |
| Heart rate (bpm) | 69 [63-78] | 63 [55-71] |
| PIP (mbar) |  | 15 [14-17] |
| PEEP (mbar) |  | 5 [5-6] |

Abbreviations: DBP, Diastolic blood pressure; GA, general anesthesia; IQR, Interquartile range; MAP, Mean arterial pressure; PEEP, Positive end-expiratory pressure; PIP, Peak respiratory pressure; SBP, Systolic blood pressure
