# Supplementary material for: Impact of arm position compared to tourniquet and general anesthesia on peripheral vein width in supine adult patients: a prospective, monocentric, cross-sectional study
Source: BMC Anesthesiol. 2024 Oct 22;24:379. doi: 10.1186/s12871-024-02765-6 (PMC11494795; doi:10.1186/s12871-024-02765-6)
Supplement: Supplementary file 2 — Supplementary Material 2. [file 12871_2024_2765_MOESM2_ESM.zip › CUVE_suppl_table_2_3_BMCA.docx]

Supplemental Tab 2 Results for cubital vein circumference

| Measuring condition | | 0°  Median [IQR] | P value / r  vs awake-NT-0° | 30°  Median [IQR] | P value / r 0° vs 30° | Max°  Median [IQR] | P value / r 0° vs max° |
| --- | --- | --- | --- | --- | --- | --- | --- |
| awake | NT, mm | 14.8 [12.6-17.8] | **-** | 15.3 [13.0-18.3] | 1.0/0.106 | 16.1 [13.5-19.1] | 0.574/0.235 |
|  | AT, mm | 16.4 [14.3-19.5] | **0.001/0.453** | 17.2 [14.6-20.9] | 1.0/0.155 | 17.6 [15.0-21.0] | 0.178/0.268 |
| GA | NT, mm | 16.2 [14.6-21.8] | **0.001/0.388** | 16.7 [14.7-21.5] | 1.0/0.095 | 17.1 [14.6-21.6] | 1.0/0.179 |
|  | AT, mm | 18.0 [16.1-23.0] | **0.001/0.837** | 18.1 [16.5-23.1] | 1.0/0.064 | 18.9 [15.8-23.8] | 1.0/0.098 |

P value / r considering p<0.05 as significant and r>0.1 a small, >0.3 a medium, and >0.5 a large strength of association. Abbreviations: AT, applied tourniquet; CuV-CI, cubital vein circumference; GA, general anesthesia; IQR, Interquartile range; OD, out of plane diameter; NT, without applied tourniquet.

Supplemental Tab 3 Results for cephalic vein circumference

| Measuring condition | | 0°  Median [IQR] | P value / r  vs awake-NT-0° | 30°  Median [IQR] | P value / r 0° vs 30° | Max°  Median [IQR] | P value / r 0° vs max° |
| --- | --- | --- | --- | --- | --- | --- | --- |
| awake | NT, mm | 4.2 [3.4-5.8] | - | 4.9 [3.9-6.0] | 1.0/0.181 | 4.9 [4.0-7.1] | 0.084/0.288 |
|  | AT, mm | 5.3 [4.1-6.2] | **0.016/0.329** | 5.2 [4.2-6.4] | 1.0/0.100 | 5.5 [4.4-6.9] | 1.0/0.202 |
| GA | NT, mm | 5.3 [4.6-6.7] | **0.001/0.405** | 6.1 [4.6-7.6] | 0.775/0.225 | 6.1 [4.9-7.6] | **0.048/0.302** |
|  | AT, mm | 6.3 [5.2-7.7] | **0.001/0.811** | 6.4 [5.5-7.8] | 1.0/0.130 | 6.3 [5.5-7.9] | 1.0/0.167 |

P value / r considering p<0.05 as significant and r>0.1 a small, >0.3 a medium, and >0.5 a large strength of association.

Abbreviations: AT, applied tourniquet; CeV-CI, cephalic vein circumference; GA, general anesthesia; IQR, Interquartile range; OD, out of plane diameter; NT, without applied tourniquet.
